# The complete mitochondrial genome of the Antarctic fairy shrimp *Branchinectagaini* Daday, 1910 (Branchiopoda, Anostraca, Branchinectidae)

**DOI:** 10.3897/BDJ.10.e94051

**Published:** 2022-10-18

**Authors:** Euna Jo, Jin-Hyoung Kim, Young Wook Ko, Sanghee Kim, Seunghyun Kang

**Affiliations:** 1 Division of Life Sciences, Korea Polar Research Institute, Incheon 21990, South Korea Division of Life Sciences, Korea Polar Research Institute Incheon 21990 South Korea; 2 College of Life Sciences and Biotechnology, Korea University, Seoul 02841, South Korea College of Life Sciences and Biotechnology, Korea University Seoul 02841 South Korea; 3 Department of Polar Sciences, University of Science and Technology, Incheon 21990, South Korea Department of Polar Sciences, University of Science and Technology Incheon 21990 South Korea

**Keywords:** *Branchinectagaini*, mitochondrial genome, Antarctica, fairy shrimp, Anostraca

## Abstract

The complete mitochondrial genome of Antarctic fairy shrimp *Branchinectagaini* Daday, 1910 was sequenced, assembled and annotated using next-generation sequencing technology. The mitogenome of *B.gaini* is circular at 15,536 bp in length, consisting of 13 protein-coding genes, 23 tRNAs, two rRNAs and two major non-coding regions. In particular, there are two tRNA^Gly^ genes and one non-coding region between these two tRNA^Gly^ genes. A phylogenetic tree was constructed using concatenated amino acid sequences of 13 protein-coding genes. It reveals that *B.gaini* is clustered with the Anostraca group within the Branchiopoda clade. This study helps us understand the evolution of Anostraca.

## Introduction

*Branchinecta Verrill 1869* (Branchiopoda, Anostraca) is a genus of freshwater fairy shrimps. The genus contains approximately 50 species, which are distributed in all continents, except Africa and Australia (Rogers 2006). Amongst them, *Branchinectagaini* Daday 1910 is the only fairy shrimp found in the Antarctic Peninsula (Peck 2004, Hawes 2009). It is also distributed in South America and subantarctic islands. As for the biogeography of *B.gaini*, passive dispersal of dormant eggs into and across Antarctica via water, winds or birds has been suggested (Hawes 2009). Despite the predominance and importance of *B.gaini* in Antarctic freshwater ecosystems (Paggi 1996), molecular studies of *B.gaini* have been insufficient. Here, we report the complete mitochondrial genome of *B.gaini* and its phylogenetic relationships within Branchiopoda, based on available mitogenome sequences.

## Material and Methods

Adult samples of *B.gaini* were collected from a freshwater pool located on Weaver Peninsula of King George Island, Antarctica (62°N and 58°E). The samples were captured using hand nets and disposable pipettes and then stored in ethanol solution. The specimens were deposited at the Korea Polar Research Institute (ID: BG-20). Total genomic DNA was extracted using the phenol/chloroform method. The quality and quantity of DNA was checked by gel electrophoresis and PicoGreen assay (Invitrogen, CA, USA), respectively. The sequencing library was prepared using a TruSeq Nano DNA kit (Illumina, CA, USA) and sequenced on an Illumina HiSeq X platform (2 × 151 bp) according to the manufacturer’s protocol. After removing adapters and low-quality sequences, the *de novo* assembly of the mitochondrial genome was conducted using the QIAGEN CLC Assembly Cell 4.2.1 programme (QIAGEN, CA, USA). Primary annotation was achieved with GeSeq (Tillich et al. 2017), followed by manual curation using Artemis (Carver et al. 2012). The mitochondrial gene map of the *B.gaini* was displayed by OrganellarGenomeDRAW (OGDRAW) version 1.3.1 (Greiner et al. 2019). For the phylogenetic analysis, General Reversible Mitochondrial (mtREV) with Frequencies (+F) model was selected as the best-fit amino acid substitution model. Maximum Likelihood (ML) tree was constructed with 1,000 bootstrap replications and the mtREV+F+G+I model (Adachi and Hasegawa 1996) using MEGA X software (Kumar et al. 2018). The mitogenome data used in the phylogenetic analysis were selected with reference to Yang and Chen (2020) and are presented in Table 1.

## Results and Discussion

**Figure 1.**
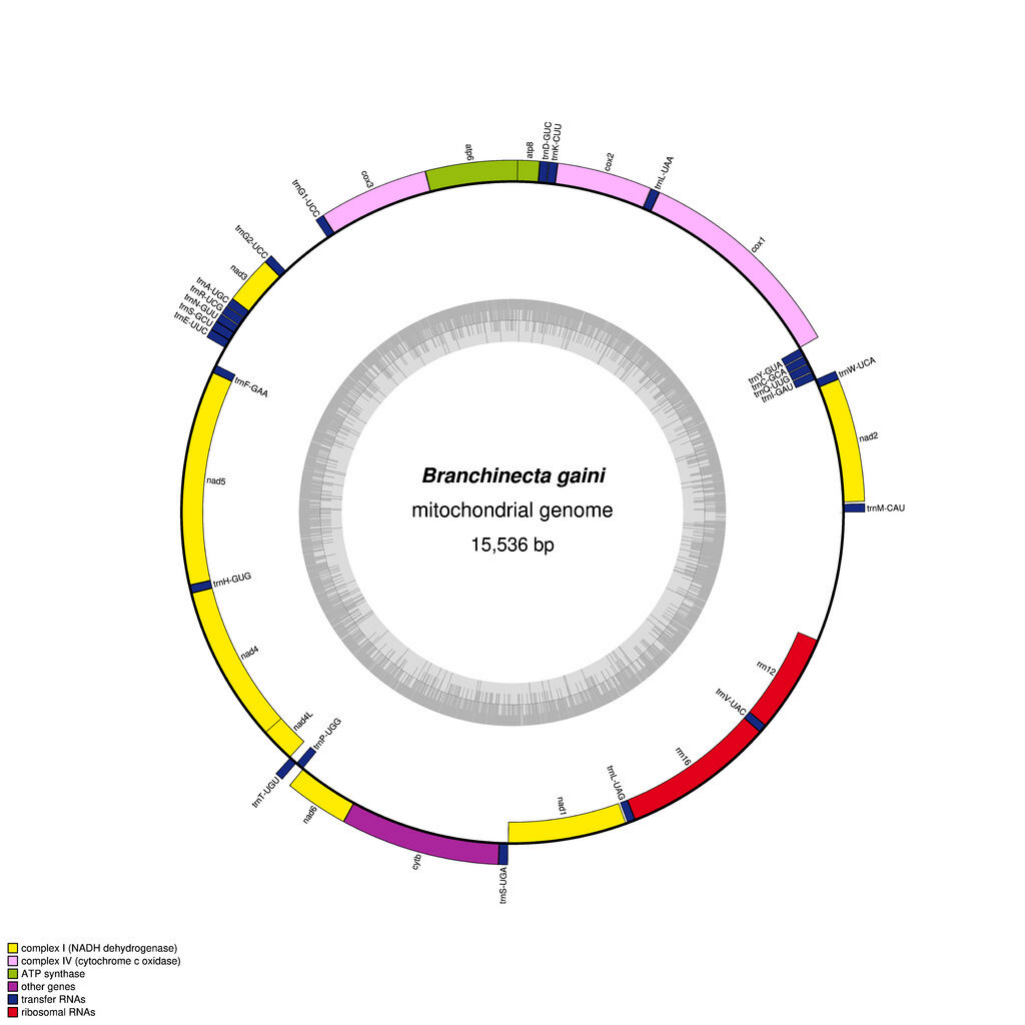
Circular map of the mitochondrial genome of *Branchinectagaini*. Genes drawn inside the circle are transcribed in a clockwise and genes drawn outside the circle are transcribed in a counterclockwise direction. The inner grey circle shows the GC content.

The complete mitochondrial genome of *B.gaini* Daday, 1910 (GenBank number: MZ265218) is 15,536 bp in length, containing 13 protein-coding genes, 23 transfer RNA genes (tRNAs), two ribosomal RNA genes (rRNAs) and two major non-coding regions (Fig. 1). Interestingly, *B.gaini* have two copies of tRNA^Gly^ genes and a 397 bp non-coding region between tRNA^Gly1^ and tRNA^Gly2^ genes. To exclude the possibility of assembly error, sequences including the two tRNA^Gly^ genes and non-coding region were reconfirmed through traditional Sanger sequencing. It was found that tRNA^Gly^ gene duplication did not appear in other anostracans or branchiopods species. The non-coding region between the tRNA^Gly^ genes was first identified in anosctacan species, although the cephalocarid crustacean *Hutchinsoniellamacracantha* was reported to have a 666 bp non-coding region between tRNA^Gly^ and nad3 genes (Lavrov et al. 2004). After more mitogenomes are revealed in closely-related species, further research is needed to explain why tRNA^Gly^ gene duplication has been occurred in *B.gaini*. The *B.gaini* mitogenome have a GC content of 35.2% and an AT content of 64.8%. Its protein-coding genes have four types of start codons (5 ATGs, 5 ATTs, 2 TTGs and 1 ATC) and three types of stop codons (6 TAAs, 2 TAGs and 5 incomplete T(AA)s).

**Figure 2.**
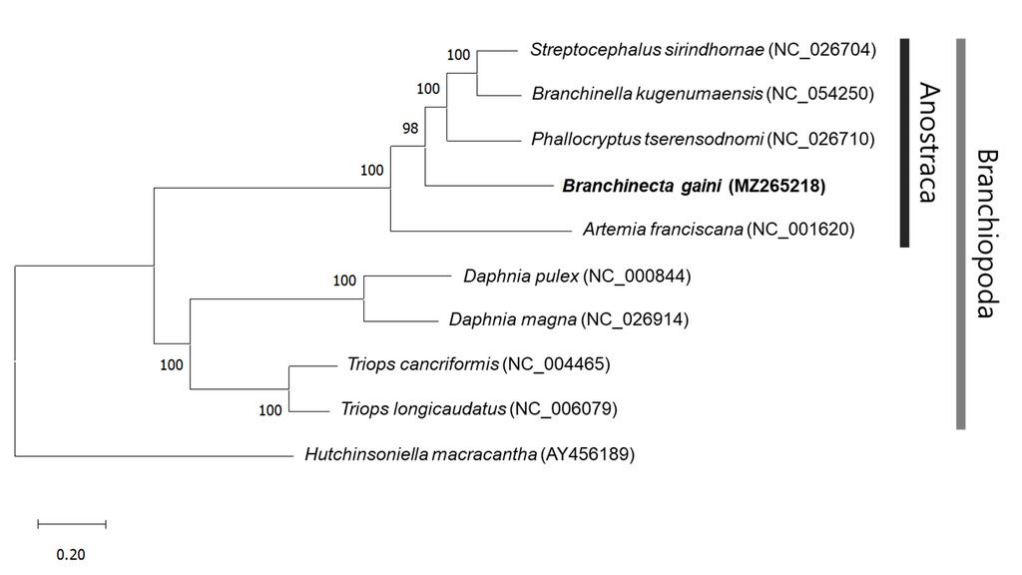
Phylogenetic tree for Branchiopoda species, based on complete mitogenome data using the Maximum Likelihood (ML) method and General Reversible Mitochondrial (mtREV) with Frequencies (+F) +G+I model implemented in MEGA X. Scientific names and GenBank accession numbers are shown for each branch. The species in this study is marked in bold. The bootstrap values are displayed on each node. The scale bar represents the number of substitutions per site.

Phylogenetic relationships of *B.gaini* with eight species within class Branchiopoda and one outgroup (*H.macracantha*) were analysed using concatenated amino acid sequences of 13 protein-coding genes (Fig. 2). The tree shows that *B.gaini* is situated in a monophyletic cluster formed by Anostraca species (*Streptocephalussirindhornae*, *Branchinellakugenumaensis*, *Phallocryptustserensodnomi* and *Artemiafranciscana*) within the Branchiopoda clade. This study reports the complete mitochondrial genome of *B.gaini*. It will help us understand the evolution of Anostraca.

## Figures and Tables

**Table 1.** List of mitogenome data used in the phylogenetic analysis.

Species name	Class	Order	Family	GenBank number	Reference
*Artemiafranciscana*	Branchiopoda	Anostraca	Artemiidae	NC_001620	Perez et al. (1994)
*Branchinectagaini*	Branchiopoda	Anostraca	Branchinectidae	MZ265218	This study
*Branchinellakugenumaensis*	Branchiopoda	Anostraca	Thamnocephalidae	NC_054250	Sun (2021)
*Phallocryptustserensodnomi*	Branchiopoda	Anostraca	Thamnocephalidae	NC_026710	Fan et al. (2016)
*Streptocephalussirindhornae*	Branchiopoda	Anostraca	Streptocephalidae	NC_026704	Liu et al. (2016)
*Daphniamagna*	Branchiopoda	Diplostraca	Daphniidae	NC_026914	Cheng et al. (2015) (unpublished)
*Daphniapulex*	Branchiopoda	Diplostraca	Daphniidae	NC_000844	Crease (1999)
*Triopscancriformis*	Branchiopoda	Notostraca	Triopsidae	NC_004465	Umetsu et al. (2002)
*Triopslongicaudatus*	Branchiopoda	Notostraca	Triopsidae	NC_006079	Cook et al. (2005)
*Hutchinsoniellamacracantha*	Cephalocarida	Brachypoda	Hutchinsoniellidae	AY456189	Lavrov et al. (2004)
